# Diversity of unique, nonmycorrhizal endophytic fungi in cultivated *Phalaenopsis* orchids: A pilot study

**DOI:** 10.1002/pei3.10146

**Published:** 2024-05-17

**Authors:** Jonathan I. Watkinson, Brenda S. J. Winkel

**Affiliations:** ^1^ Department of Biological Sciences Virginia Tech Blacksburg Virginia USA; ^2^ Fralin Life Sciences Center Virginia Tech Blacksburg Virginia USA

**Keywords:** ascomycota, endophytes, horticultural, phytochemical, screening method

## Abstract

Orchids comprise one of the largest, most diverse, and most broadly distributed families of flowering plants and contribute significantly to habitat biodiversity. One key aspect of orchid growth and development is the formation of mycorrhizal symbioses with compatible endophytic fungi, which are maintained throughout the life of the plant. Substantial efforts to identify the fungi that form mycorrhizal symbioses across a range of orchid species have often also uncovered numerous nonmycorrhizal, endophytic fungi. These fungi could also have significant effects on orchid growth and development and are beginning to be analyzed more closely, particularly in wild species. The role of endophytic fungi in the production, distribution, and continued growth by the hobbyist of orchids is not known. As an initial step toward characterizing nonmycorrhizal endophytic fungi associated with cultivated orchids, we undertook a survey of fungi residing within roots of *Phalaenopsis* plants growing in home environments. Sequence analysis of ITS regions amplified from total DNA isolated from roots allowed rapid identification of endophytic fungi to the class level and may offer a useful initial screening method for beneficial species, for example, in horticultural settings. ITS‐PCR sequences subsequently obtained from individual fungi cultured from surface‐sterilized orchid roots corroborated the findings of the initial screen, while also providing a more complete characterization of the array of fungal taxa that were present. Although lower in diversity than has been reported for orchids growing in the wild, these endophytes have the potential to substantially enhance the growth and disease resistance of horticultural orchids.

## Graphical abstract

We present a preliminary study aimed at using a cost‐effective approach to determine fungi present in Phalaenopsis roots at the end of the production pipeline. Significantly, the work identified the presence of unique fungal species in roots of the different Phalaenopsis plants and provided a simple screening technique that could be applied at any point in the production pipeline. The work provides a foundation for more detailed studies into analyzing the effects of cultivation practices on the community of endophytic fungi in orchids.

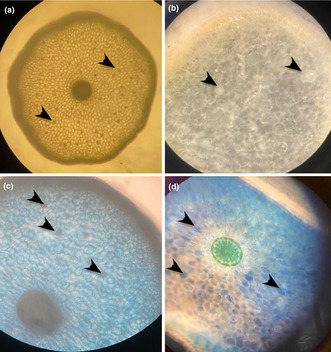

## 1 INTRODUCTION

Orchids comprise one of the largest and most diverse families of flowering plants, with an estimated 31,000 species distributed across almost every continent (Fay, 2016; Fay et al., 2015; Hinsley et al., 2018). One critical aspect of orchid biology is a reliance on mycorrhizal fungi (Rasmussen & Rasmussen, 2008). Orchid seeds are devoid of endosperm, the nutritive tissue that normally drives germination, and therefore rely on outside sources of energy to spur this stage of plant growth (Rasmussen et al., 2015). In nature, orchid seed germination is promoted by interactions with specific species of fungi, termed orchid mycorrhizal fungi (OMF). The requirement for OMF to initiate germination was initially demonstrated in the early 1900s (Selosse et al., 2017) and it is now generally accepted that OMF are essential for orchid germination in situ (Alghamdi, 2019; Meng et al., 2019; Rasmussen et al., 2015). Mutualism between fungi and young orchid plantlets has also been demonstrated and there is substantial evidence that older plants depend on mycorrhizae for growth and survival, although much remains to be explored in this area (Bunch et al., 2013; Cameron et al., 2006; de la Rosa‐Manzano et al., 2014; McCormick et al., 2018). The presence of OMF then seems to be a crucial factor in the persistence of orchids in a variety of habitats, which could make these plants particularly vulnerable to factors that negatively impact the sustainability of fungal populations.

Orchids also contain numerous species of endophytic fungi, both in roots and other tissues, which are nonmycorrhizal (orchid nonmycorrhizal fungi or ONF), although these are much less well studied (Cevallos et al., 2018; Li et al., 2021; Ma et al., 2015; Selosse et al., 2022; Sisti et al., 2019; Suárez & Kottke, 2016). While the fungi that form the orchid‐mycorrhizal relationship belong to several families within the Basidiomycota, most notably (but not limited to) *Serendipitaceae* (Sebacinales), *Ceratobasidiaceae*, and *Tullasnellaceae* (Favre‐Godal et al., 2020), ONF exhibit a much higher species diversity, distributed among some 110 genera, the majority of which are Ascomycetes (Li et al., 2021). Many of these ONF are well‐known endophytes of other plants, where these fungi have received significant attention as a source of bioactive compounds, including novel antimicrobials (Caruso et al., 2020). Although ONF are not required for orchid germination and seedling growth, there is a growing awareness that these fungi may facilitate germination and that they are important for growth and survival of the mature plant (Bungtongdee et al., 2019; Li et al., 2021; Pant et al., 2017). There is also mounting support for the hypothesis that a diverse array of endophytic fungi has allowed for the evolution of mycorrhizal fungi through the adaptation of preexisting ecological functions and structures (Ma et al., 2015; Selosse et al., 2022; Shah et al., 2019). Continued research into the full complement of endophytic fungi present within orchid roots is essential for understanding the complexity of interactions necessary for growth, development, and survival of orchids as well as other plant species.

In addition to being important in numerous and varied ecosystems, orchids have become popular as houseplants, particularly species and hybrids of the genera *Phalaenopsis*, *Cymbidium*, and *Dendrobium* (Hinsley et al., 2018). In the US, *Phalaenopsis* is the most popular orchid, with annual production estimated to be in millions of units and revenue in the hundreds of millions of dollars. The majority of these plants are produced in vitro using plant tissue culture techniques under sterile conditions before being transplanted into suitable growth media that typically contain bark, charcoal, perlite, and/or sphagnum moss (Sanghamitra et al., 2019). Most manufacturers do not sterilize their bark mixes so that although the orchids are produced aseptically, they are exposed to fungi and bacteria upon transfer to the growth medium. The microbes that cultivated orchids acquire will thus depend on the source of the potting mix as well as its makeup (e.g., fir bark, pine bark, or sphagnum). Furthermore, the growing facility, the retail outlet, and the orchid's subsequent locale(s) all have the potential to influence the array of ONF that are ultimately associated with cultivated orchids.

Although a number of studies have begun to examine ONF in cultivated orchids, little has been done to examine the essential role of fungi in the successful transition and maintenance of orchids in greenhouses and other artificial environments (Dearnaley et al., 2012). Huang et al. (2014) identified 500 OTUs across a range of fungal phyla in commercially grown *Phalaenopsis* plants, although the primary purpose of this study was to determine the effectiveness of different primer sets in generating OTUs. Similarly, Zhu et al. (2022) examined endophytes of *Dendrobium catenatum* and showed that microbial communities differed depending on the site of cultivation, which included trees, rocks, and plastic pots, aiming to optimize the accumulation of phytochemicals associated with the use of this plant as traditional medicine. While these studies provide some insight into the role of endophytic fungi in cultivated orchids, there remains a large knowledge gap centered around the dynamics of endophytic fungi during the production, distribution, and maintenance of cultivated orchids. Examination of endophytic fungi in cultivated *Phalaenopsis*, which represents the largest share of the horticultural orchid trade in the U.S., will provide relevant information for producers and consumers alike. To this end, we used two complementary approaches to perform a preliminary survey of fungi from several cultivated *Phalaenopsis* hybrids from different locations. This pilot study demonstrates the feasibility of using cost‐effective screening techniques to generate a snapshot of endophytic fungi present at the end of the production pipeline.

## 2 MATERIALS AND METHODS

### 2.1 Plant materials

Orchid plants were purchased over a period of 10 years from a variety of retail outlets and maintained in four different residences. Plants used consisted of five *Phalaenopsis* cultivars (3–10 years old). Most were grown in plastic pots containing a mix of bark, perlite, and charcoal and re‐potted as the media decayed (*Phalaenopsis* 1, 2, and 5). One was grown in sphagnum moss in a plastic pot (*Phalaenopsis* 3) and another was mounted on sphagnum moss in a wooden box (*Phalaenopsis* 4). Plants were grown following common home orchid growing instructions: watered as media dried out and periodically fertilized. Roots for analysis were selected based on the criteria that they had actively growing root tips, were in contact with the medium, and had pigmented regions that could be indicative of mycorrhizal colonization (Zettler & Corey, 2018). Two roots (denoted a and b below) were excised from each plant with a clean razor blade, placed in sealable plastic bags, and kept at 4°C prior to processing (typically the same day). Each root sample was processed separately, with half used for DNA extraction and half used for fungal cultivation.

### 2.2 Cultivation of fungal endophytes

Root samples were cut into 1 cm sections and surface sterilized for 10 min in a 10% bleach solution (commercial 6.25% sodium hypochlorite). This was followed by immersion for 2 min in 95% ethanol and then transfer to sterile Milli‐Q water. The root sections were placed into sterile petri dishes and further sliced into 1–2‐mm‐thick sections with sterile razor blades. These sections were transferred to plates containing phytone yeast extract medium (PYE, Currah et al., 1997) which we modified to contain 5 g (vs. 40 g) L^−1^ sucrose (mPYE) since reduced sugar has been shown to favor isolation of slower growing fungal species (Zettler & Corey, 2018). Chloramphenicol (50 mg L^−1^) and streptomycin (30 mg L^−1^) were added to control bacterial growth. Plates were incubated at 28°C for 1–3 days. Fungi emerging from root sections were subcultured individually onto mPYE plates, using a sterile needle to isolate hyphae from the edge of the growing colony. All manipulations of fungal cultures were carried out in a biological safety cabinet.

### 2.3 DNA isolation

Orchid root samples were surface sterilized for 10 min in 10% bleach, rinsed in sterile water, and then ground to a powder in liquid nitrogen. DNA was isolated from approximately 50 mg of ground plant tissue using a DNeasy Plant Kit (QIAgen). Typical yields were 0.5–1.0 μg of DNA in a volume of 50 μL. DNA concentration and quality were determined using a DeNovix DS‐11 Nanodrop spectrophotometer.

DNA was extracted from fungal hyphae obtained from mPYE plates using either the DNeasy Plant Mini Kit (Qiagen) or the E.Z.N.A. Extraction Kit (Omega Bio‐tek). Tissue was ground in 1.5 mL microfuge tubes with 40 μL of extraction buffer using a mini‐plastic mortar. After grinding, another 350 μL of extraction buffer was added and the samples were heated and processed further according to the kit manufacturer's protocol. DNA was extracted from commercially available *Agaricus bisporus* (i.e., white mushroom, purchased from a local grocery store) for use as a positive control.

### 2.4 PCR

Amplification reactions of 25 μL were performed using approximately 100 ng of template DNA, MyTaq™ Red Mix (Bioline), and either the ITS‐OF1/ITS4OF primer set designed by Taylor and McCormick (2008) or ITS3 (White et al., 1990)/ITS4OF as per Jacquemyn et al. (2015). The final primer concentrations were 200 nM. Thermocycler parameters for both primer sets consisted of an initial step at 95°C for 5 mins, followed by 35 cycles of 95°C (30 s), 52°C (30 s), and 72°C (30 s), and then a final extension at 72°C for 5 min. PCR products (5 μL) were analyzed on a 1% agarose gel. For samples that showed a band of the expected size, the remainder of the reaction was purified using a QiaQuick PCR clean‐up kit (Qiagen) according to the manufacturer's directions, followed by elution in nuclease‐free water. Sanger sequencing was performed using either ITS4OF or ITS3 at the Virginia Tech Fralin Life Sciences Institute Genomics Sequencing Center. All sequences were searched against the GenBank ITS Fungal Type Collection using BLAST through the National Center for Biotechnology Information web interface. Matches with the highest bit score and lowest e‐values were used as the most likely identity for each fungal isolate.

### 2.5 Microscopy

Root sections were examined using an Olympus SZX12 dissecting microscope at 45–60× magnification. Roots were illuminated from either above or below and photographed using an iPhone11. Trypan blue staining of root sections was accomplished according to Rains et al. (2003). Briefly, roots were placed in hot 10% KOH for 1 h. The material was then rinsed in acidified water and placed in Trypan Blue solution (GIBCO), heated briefly at 90°C, and then left at room temperature for 1 h. Roots were destained in water for 1 h, rinsed in deionized water, and then immediately visualized and photographed.

## 3 RESULTS

### 3.1 Identification of fungi from DNA isolated from whole roots

**FIGURE 1:**
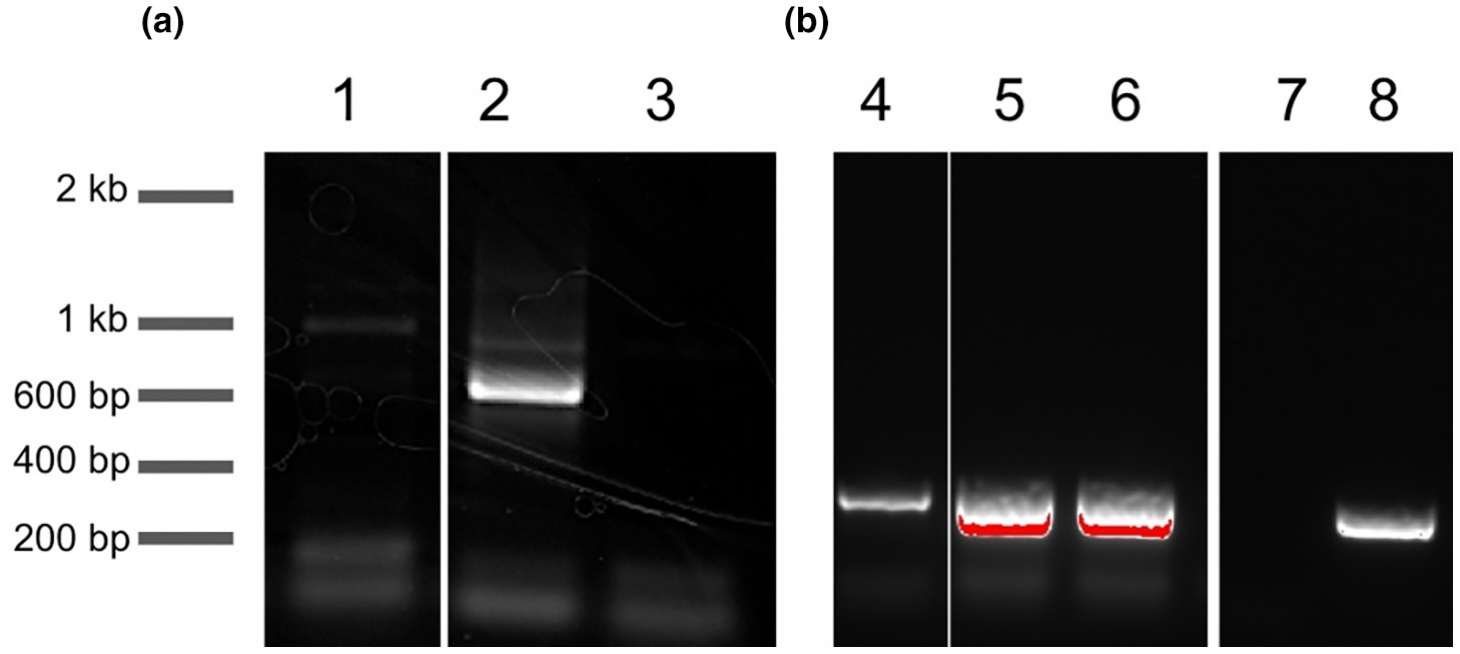
PCR amplification of fungal ITS from orchid root and root fungal isolate DNA extracts. PCR was performed with the ITS1OF/ITS4OF (a) or ITS3/ITS4OF (b) primer pairs. Samples include *Phalaenopsis* root DNA (1, 4), individual fungi isolated from Phalaenopsis roots (5, 6), *Agaricus bisporus* DNA positive control (2, 8), no DNA negative control (3, 7).

To begin to understand how the fungal microbiome of cultivated orchids may contribute to survival and adaptation during the horticultural production pipeline, we undertook a pilot study of endophytic fungi associated with roots of *Phalaenopsis* plants that had been grown by several different consumers. We first tested the feasibility of using PCR to identify the most abundant fungal species associated with these samples as an efficient, cost‐effective, initial assessment. DNA of high quality was isolated from two surface‐sterilized root samples from each of the five *Phalaenopsis* plants. PCR was performed using primers designed to amplify the internal transcribed sequences of basidiomycetous fungal genomic DNA, but not plant or bacterial DNA (Taylor & McCormick, 2008). Reactions performed with the ITS1OF/ITS4OF primer pair produced amplicons of the expected size (which ranges from 500 to as long as 900 bp and Figure 1a); however, the production of amplicons was inconsistent. In contrast, the ITS3/ITS4OF primer pair (Jacquemyn et al., 2015; Waud et al., 2014) reliably generated amplicons of the expected 300–400 bp size from these same tissues (Figure 1b). The ITS2 region amplified by this primer set has become a standard for barcoding in fungi, particularly for Ascomycota and Basidiomycota (Guillen‐Otero et al., 2023; Xu, 2016).

Sanger sequencing of the cleaned PCR reactions from total DNA derived from the orchid roots produced traces with multiple, overlapping reads in a single reaction, consistent with the presence of multiple amplicons in each PCR. Despite the overlapping traces, the consensus sequence generated by the analysis software (4Peaks) produced a significant match when subjected to BLASTn against the ITS Fungal Type collection. Table 1 shows the top BLASTn hit for sequences obtained from individual root samples using the ITS3/ITS4OF primer set. Unfortunately, the limited amount of DNA from *Phalaenopsis* 1 coupled with the failure of the OF fungal primer set meant that we were unable to obtain sequence data for the roots of this plant. For several samples tested, the top match was to Ascomycetous fungi, including *Coniochaeta* and *Chloridium*. Other samples generated top matches to a *Fusarium* sp., and to the Basidiomycete yeast, *Rhodotorula* sp., and an uncultured *Serendepitaceae*. These results suggest that the consensus sequence generated by this method can identify the presence of nonmycorrhizal species in these samples.

**TABLE 1:** Identities of abundant fungal species associated with horticultural *Phalaenopsis* roots based on ITS sequence analysis of total genomic DNA^a^.

Source	Putative identity^b^	GenBank ID	Bit score	E value	Subdivision	Class
*Phalaenopsis* hybrid 2 root a	*Rhodotorula* sp.	OR194161	560	8 e−160	Basidiomycota	Pucciniomycotina
Root b	*Coniochaeta* sp.*	OQ565311	774	0	Ascomycota	Sordariomycetes
*Phalaenopsis* hybrid 3 a	*Coniochaeta* sp*	OQ565313	431	3 e−121	Ascomycota	Sordariomycetes
b	No match					
*Phalaenopsis* hybrid 4 root a	Uncultured Serendepitaceae	*NS*	61	2 e−04	Basidiomycota	Agaricomycetes
Root b	*Chloridium* sp.	OQ565316	470	7 e−133	Ascomycota	Sordariomycetes
*Phalaenopsis* hybrid 5 a	*Fusarium* sp.		200	7 e−47	Ascomycota	Sordariomycetes
b	Same as above					

^a^ Identity determined by comparison of sequences identified against the fungal type nucleotide collection at NCBI using BLASTn.

^b^ Putative identities of genera are presented based on the highest bit‐score match. Asterisk indicates isolates that matched the same accession in the NCBI database.

### 3.2 Microscopic analysis of fungi in roots

**FIGURE 2:**
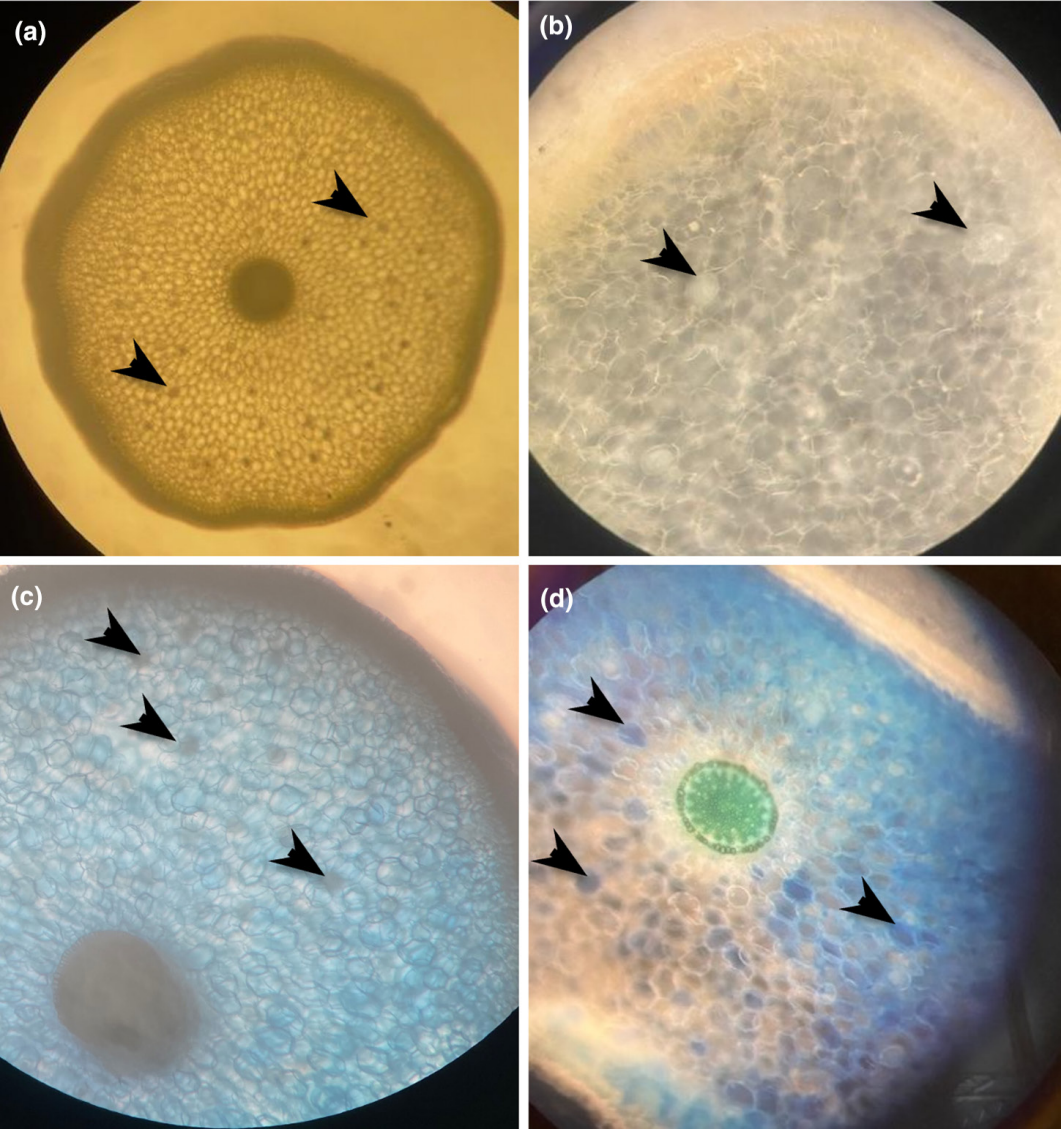
Staining of *Phalaenopsis* root sections to identify putative locations of fungi. Root sections were cultured on mPYE fungal isolation medium for 2 days (a, b) or left uncultured and stained with trypan blue (c, d). Arrows point to dense or dark regions in roots placed on fungal isolation medium (a, b) or to cells showing trypan blue staining (c, d). A is 10× magnification, b is at 60×, c and d at 40× magnification.

The presence of OMF is often associated with the formation of intracellular fungal coils called pelotons. Because the initial screen did not identify known mycorrhizal fungi, a visual examination of the roots was undertaken to determine whether fungal structures associated with OMF could be identified or, in accordance with the initial screen, if fungi were present. To this end, root sections were observed under a dissecting microscope illuminated from above and/or below the stage. Fungi seemed apparent as dark or dense regions in some cells (arrows in Figure 2a,b) as has been noted by others (see, e.g., Sisti et al. [2019] and Weerasuriya et al. [2022]). This was confirmed with Trypan blue staining, which is widely used to identify the presence of mycorrhizal fungal structures in plant tissues (Phillips & Hayman, 1970), showing the presence of dense, darker blue regions within some cells (Figure 2c,d). It was not possible to determine if these were fungal peletons, and therefore mycorrhizal status could not be confirmed. However, the microscopic analysis provided visual evidence that the orchid roots contained structures that could be identified histochemically as fungi.

### 3.3 Identification of fungi cultivated from root sections

In an effort to build on the preliminary analysis, individual fungal species were cultivated from the same roots obtained from the five *Phalaenopsis* plants. Fungi were recovered by placing sections of sterilized orchid roots onto mPYE fungal isolation medium. Hyphal outgrowth was typically observed within 24–48 h, although in some cases fungi did not appear until after 5 days and several showed no outgrowth even after 14 days or more (data not shown). Most fungi grew slowly and the root pieces remained healthy. All of these fungi, with a few exceptions, were subcultured. In two instances, fungi rapidly grew over the root pieces and overtook the isolation plate, turning the root section necrotic. The rapid rate of growth of these isolates prevented the efficient passage of these two cultures and, along with concerns about their aggressive nature, these were omitted from further analysis. It is possible that these were latent‐pathogen endophytes that were able to rapidly grow and outcompete other fungi under the ideal culture conditions.

Fungi were subcultured onto fresh mPYE medium for the selection of individual strains. The resulting samples were then used for DNA isolation and subsequent PCR using the ITS3/ITS4OF primer pair, as above. PCR samples that exhibited single bands on agarose gels were selected for Sanger sequencing. In contrast to the results with ITS fragments amplified from total DNA samples derived from plant roots, PCR products from the fungal isolates produced high‐quality sequence traces with reliable reads on the order of 300 nt. These were again analyzed using BLASTn against the NCBI ITS fungal‐type database. In all, 30 fungi were identified in the orchid roots. Several isolates from a single root turned out to be duplicates and some samples were recalcitrant to PCR amplification and sequencing. Table 2 shows the putative identity of 17 unique fungal species isolated from the five *Phalaenopsis* hybrids. Consistent with the findings from the preliminary ITS‐PCR analysis of DNA samples from orchid roots (Table 1), the cultivated fungi were largely identified as Ascomycetes, with the exception of two Basidiomycetous yeasts. Within the Ascomycetes, fungi were largely found in different orders of the Sordariomycetes. Putative identification indicated that these included three species of *Fusarium*, plus species of *Chloridium*, and *Coniochaeta*, as well as *Thermavioloides*, *Hyphodiscus*, and *Hypomontagnella*. These genus‐level identifications were aligned with the predictions of the preliminary PCR screen. Altogether, these results indicate that a diverse array of ONF is present in roots of cultivated orchids, despite this group of fungi being much less well studied than the mycorrhizae.

**TABLE 2:** Identities of fungal species cultured from roots of horticultural orchids based on ITS sequence.^a^

Source/GenBank ID	Score/ Evalue	Putative identity^b^	Subdivision	Class	Documented associations
*Phalaenopsis* hybrid 1					
OQ565315	564/3e−156	*Hypomontagnella barbarensis*	Ascomycota	Sordariomycetes	Fungal endophyte known to produce antifungal polyketides (Rahayu et al., 2021)
*Phalaenopsis* hybrid 2					
OQ565308	869/0	*Pleurostoma richardsiae*	Ascomycota	Sordariomycetes	Typically associated with dieback in agronomic crops
OQ565316	595/1 e − 165	*Chloridium viriscens*	Ascomycota	Sordariomycetes	Found as an endophyte in Neem and produces a napthaquinone (Kharwar et al., 2009)
OQ565306	1018/0	*Phaeacremonium tardicrescens*	Deutoromycota	Hyphomycetes	Wilt in grape; Lactone producing endophyte of Senna (Silva et al., 2017)
*Phalaenopsis* hybrid 3					
OQ565310	577/4 e−165	*Fusarium foetens*	Ascomycota	Sordariomycetes	Beneficial, harmful or neutral endophytes of many plants; numerous references
OQ565325	577/4 e−165	*Coniochaeta* sp.	Ascomycota	Sordariomycetes	Diverse fungal metabolites with antimicrobial properties (Sugijanto et al., 2009)
OQ565326	693/0	*Rhodotorula mucilaginosa*	Basidiomycota	Pucciniomycotina	Used as a biocontrol for molds on apples (Qian et al., 2020)
OQ565335	582/8e−132	*Exophiala elyosperma*	Ascomycota	Chaetothyriomycetes	Endophyte of *Bletilla striata* (Zeng et al., 2021)
*Phalaenopsis* hybrid 4					
OQ565318	592 /1 e−164	*Thermothielavioides*	Ascomycota	Sordariomycetes	Polysaccharide degrading enzymes (Madathil et al., 2021)
OQ565319	651/0	*Colacogloea cycloclastica*	Basidiomycota		Mycoparasites (Sampaio et al., 2011)
OQ565320	569/6 e−158	*Phialocephala* sp.	Ascomycota	Leotiomycetes	Common root endophyte species (Grünig et al., 2008)
OQ565322	534/2 e−152	*Penicillium griseolum*	Ascomycota	Eurotiomycetes	Many diverse effects, phosphate mobilization, antibacterial (Adhikari & Pandey, 2019; Caruso et al., 2020)
OQ565328	497/3 e−141	*Taloromyces francoae*	Ascomycota	Eurotiomycetes	Mangrove endophyte producing various secondary metabolites (Nicoletti et al., 2018)
OQ565330	561/1 e−155	Exobasidiomycete	Basidiomycota	Ustilaginomycotina	
*Phalaenopsis* hybrid 5					
OQ565332	528/2 e−153	*Fusarium falciforme*	Ascomycota	Sordariomycetes	Beneficial, harmful or neutral endophytes of many plants (Bilal et al., 2018)
OQ565333	409/1 e−114	*Hyphodiscus brevicollaris*	Ascomycota	Sordariomycetes	Endophyte of Dendrobium nobile associated with fatty acid production. (Zhao et al., 2023)
OQ565331	601/3 e−172	*Sporothrix eucalyptigena*	Ascomycota	Sordariomycetes	Mangrove associated endophyte (Osorio et al., 2016)

^a^ Identity determined by comparison of sequences identified against the fungal type nucleotide collection at NCBI using BLASTn.

^b^ Putative identities of genera are presented based on highest bit‐score match.

## 4 DISCUSSION

The relationship between orchids and compatible fungi is crucial to the establishment of and persistence of orchid populations in the wild. Many studies have been conducted to successfully identify OMF in a variety of orchid species. However, more often than not, these studies also identify many other fungal species, raising the possibility of a more complex role for fungi in orchid growth and development (Cevallos et al., 2018; Li et al., 2021; Ma et al., 2015). The functional aspects of these nonmycorrhizal fungal species are only just beginning to be explored in detail in orchids growing in the wild, including whether their impact on plant growth could act synergistically with or counter to that of OMF (Pant et al., 2017; Shah et al., 2019). Even less is known about the nature of fungi in roots of cultivated orchids, which are typically propagated aseptically and then placed in an artificial growth medium that aims to emulate natural habitats. Considering that *Phalaenopsis* alone garners millions of dollars in revenue each year for growers, understanding the role of endophytic fungi in cultivated orchids has substantial potential benefits in the production pipeline. A recent study provided evidence that cultivation influences the microbiome as *Dendrobium catenatum* grown in pots had a different fungal community than plants growing on trees or rocks in the same location (Zhu et al., 2022). However, cultivated orchids face a range of factors that could influence the fungal microbiome including the location in which they were propagated, the medium they are growing in, the age of the plant, the cultural conditions provided by the hobbyist grower, and even the choice of growth container. These varying conditions could have a significant impact on the types of fungi and even the diversity of fungi present in roots, which could have implications for the persistence of these plants under cultivation, including in indoor settings. As an initial step, we undertook a survey of fungi in several different *Phalaenopsis* hybrids to determine the feasibility of cost‐effective screening methods, the types of fungi present in roots, and the possible roles of these organisms on the growth and survival of horticultural orchids.

### 4.1 ONF are present in roots of horticultural orchids

We first tested a rapid, cost‐effective way to identify fungi in roots of cultivated *Phalaenopsis* using ITS‐PCR of root total DNA extracts using fungal‐specific primers. This initial approach successfully generated amplicons from most of the sampled roots and provided a snapshot of the fungi that were present. Three of the five samples tested generated matches to Sordariomycete fungi with preliminary identification of *Coniochaeta*, *Chloridium*, and *Fusarium*; two others had matches with the Basidiomycetes, *Rhodotorula*, and an unknown *Serendipitaceae*. Intriguingly, two of the plants appeared to have different fungi in each of the tested roots, suggesting that individual roots may harbor somewhat different arrays of fungal species. It must be noted that while these sequences represent a consensus of the multiple fungi present and thus bias to more prevalent fungi, this approach does provide a class‐level overview of these organisms, as validated by subsequent sequence analysis of fungi cultivated from these samples. Microscopic analysis also indicated the presence of fungi internal to these roots.

### 4.2 Identified ONF are known endophytes with potential beneficial roles

Culturing fungi associated with orchid roots prior to ITS sequence analysis was another viable, economical approach and allowed for a more robust outcome. Up to six individual fungal taxa were identified per root sample using this method (Table 2). Most roots generated a number of isolates, however, some were duplicates and some failed to generate PCR amplicons after DNA extraction and were not included. Several potential latent‐pathogen endophytes that overgrew the roots in culture were also omitted from the analysis. This approach is more comprehensive than the conventional method of culturing orchid root‐associated fungi from pelotons, which precludes identification of nonmycorrhizal fungi. Overall, the species abundance was low, considering that some orchids growing in the wild can harbor up to 70 species of fungi (Kohout et al., 2013); this may reflect a fundamental difference between wild‐grown and cultivated orchids and/or the presence of un‐culturable or slow‐to‐cultivate fungal species within these roots. Again, this may bias the results, but does provide a preliminary snapshot of what is present in the roots. Several of the identified fungi were the same as or similar to those in the initial screen (*Fusarium*, *Coniochaeta*, *Chloridium*, and the Basidiomycete *Rhodotorula*), indicating that sequencing of PCR products derived from total DNA offers a simple and low‐cost method for the initial assessment of abundant fungi present in orchid roots.

The bulk of the identified fungi belonged to the Sordariomycete class of Ascomycota. Although the Sordariomycetes are a large class of fungi with diverse ecological roles, the species identified here belong to genera with members that are known endophytes of various other plant species. For example, *Chloridium* is a known endophyte of mangroves (*Rhizophora*) and of the neem tree (*Azadirachta indica*; Kharwar et al., 2009). Likewise, *Lecythophora* (*Coniochaeta*) species have been found as endophytes in numerous tropical species (Sugijanto et al., 2009, 2011). Two other Sordariomycetes, *Fusarium* and *Hyphodiscus*, are known endophytes of other orchids (Zhao et al., 2023). Other Ascomycetous fungi included species of *Penicillium* and *Phialocephela*, which have been found in numerous plant species, as well as *Talaromyces*, an endophyte of mangroves and *Exophilia*, another orchid endophyte (Grünig et al., 2008; Nicoletti et al., 2018; Osorio et al., 2016). Of the two Basidiomycete fungi, one, *Rhodotorula* sp., is a common plant endophyte (Qian et al., 2020). Thus, cultivation of fungi from roots gave a broader view of the endophytes present in these tissues, demonstrating that they are similar to those known to associate with other plant species.

The question that arises with the identification of the diverse group of endophytes found in *Phalaenopsis* roots is what, if any, benefit they provide to cultivated orchids. A plethora of potential benefits have previously been ascribed to a number of these species. *Chloridium*, for example, produces napthoquinone with antimicrobial properties (Kharwar et al., 2009). *Lecythophora* produces lecythomycin, a putative antimicrobial compound that is believed to protect the host plant from pathogen attack (Sugijanto et al., 2009, 2011). Similarly, species of *Hypomontagnella* have been found to produce antifungal polyketides (Rahayu et al., 2021)*. Fusarium* species, which were found in three of the five plants tested, are known to produce an abundance of compounds with diverse biological effects across a range of plant species (Bilal et al., 2018). *Fusarium* extracts have also been shown to be important in orchid growth and development, enhancing in vitro growth of *Dendrobium moniliforme* (Shah et al., 2019) and stimulating germination (Li et al., 2021). Similarly, *Hyphodiscus* is associated with the production of specific fatty acids that are associated with growth promotion in *Dendobium nobile* (Zhao et al., 2023). A number of the non‐Sordariomycete fungi identified in this study also have potential beneficial attributes. *Taloromyces* and *Phialocephela*, for example, are known to produce a range of secondary metabolites with purported benefits for the host plant (Grünig et al., 2008; Nicoletti et al., 2018). *Penicillium* species also produce a range of compounds, some with antimicrobial activity that could protect plants from pathogens (Caruso et al., 2020). Other studies indicate that endophytic *Penicillium* plays a role in phosphate mobilization (Adhikari & Pandey, 2019). As it is likely that phosphorous is limiting in many orchid habitats (Hou et al., 2020), these fungi could be crucial to sustaining plant growth and development. Yet other fungi, in particular the basidiomycetous yeast *Rhodotorula*, have the ability to produce bioactive compounds and serve as biocontrol agents against pathogenic fungi and bacteria (Qian et al., 2020; Safitri et al., 2021). The presence of these endophytic fungi in cultivated Phalaenopsis may thus be related to a protective and beneficial role of fungal endophytes in orchids growing both in the wild and in artificial environments.

It is important to note that some of the identified species are known pathogens in other plants; *Pleurostoma richardsieae*, for example, is associated with dieback in *Vitis vinifera* (Carlucci et al., 2015). *Phaeoacrimonium* is associated with wilt disease of grapes and olives (Carlucci et al., 2015; Crous et al., 1996). *Fusarium* is also a potent pathogen of numerous plants, the most well‐known being *Musa* (Czislowski et al., 2021). Intriguingly, these last two are known as beneficial endophytes in other plants (*Phaeoacrimonium* in *Senna* and *Fusarium* in orchids), suggesting a mechanism of role switching that may depend on growing conditions.

Although the initial PCR screen generated a partial match to a member of the *Serendipitaceae*, a known mycorrhizal family, no other OMF were identified among the cultivated fungi, despite being readily cultured (Zettler & Corey, 2018). This could indicate a general absence of OMF in the collected samples, and therefore that OMF fungal inoculum was not present either in the production pipeline or the home environments where these orchids were grown. This is surprising, as OMF present in the environment have been shown to avidly colonize the roots of horticultural orchids, for example when growing on urban trees (Izuddin et al., 2019). That the situation may be different for orchids growing indoors is an intriguing possibility that remains to be confirmed. Future studies will use a metagenomics approach to provide a more comprehensive assessment of the fungi inhabiting the roots of cultivated orchids.

## 5 CONCLUSIONS

A pilot study of the diversity of fungi in the roots of home‐grown orchids identified numerous species of ONF with potential positive roles for the plant host. For this initial examination, techniques for fungal identification included using PCR to amplify fungal sequences from sterilized root sections or cultivating individual fungi directly from roots and identifying each isolate based on Sanger sequencing of the amplified ITS. Direct PCR of DNA isolated from intact roots proved to be a useful approach for generating putative identities of the fungi present that could serve as an initial screening method, for example, for commercial growers. Culturing of fungi from these roots followed by identification based on ITS‐PCR corroborated the identifications made in the initial screen and provided evidence for the presence of numerous additional species. These experiments indicated that roots from the orchids that were sampled harbored a number of ONF, particularly in the *Sordariomycete* class. Overall, individual *Phalaenopsis* plants harbored unique fungal constituents, suggesting that the environment and/or cultural practices influenced the fungal biome. Intriguingly, the fungi that were present largely represented species that are known endophytes of many different plants and that provide benefits in the form of protection from pathogens through the synthesis of key metabolites as well as in nutrient mobilization. Although our preliminary findings indicate that cultivated orchids harbor a relatively low overall diversity of fungal species, the identified fungi are expected to play an essential role in the growth and survival of these plants in nonnative environments. A deeper understanding of the complexity and dynamics of ONF and OMF in cultivated and wild orchids, including by extending these studies using next‐generation sequencing methods for high‐resolution unbiased characterization of these fungal communities, should provide important new insights into the contributions of the fungal microbiome to plant adaptability and resilience.

## FUNDING INFORMATION

This work was funded by internal funds from The Fralin Life Sciences Center and the Department of Biological Sciences at Virginia Tech.

## CONFLICT OF INTEREST STATEMENT

The authors state that there is no conflict of interest.

## Data Availability

All sequence data has been submitted to NCBI and accession numbers are present in the manuscript. All other data will be made available when the manuscript is accepted.
